# Enhancing targeted doses: Low‐energy photon lipiodol‐enhanced radiotherapy (LEPERT) for liver cancer patients

**DOI:** 10.1002/acm2.14578

**Published:** 2024-11-29

**Authors:** Daisuke Kawahara

**Affiliations:** ^1^ Department of Radiation Oncology Graduate School of Biomedical Health Sciences Hiroshima University Hiroshima Japan

**Keywords:** dose enhancement, lipiodol, radiotherapy

## Abstract

**Objectives:**

This study proposes a novel approach, “Low‐energy photon Lipiodol‐Enhanced Radiotherapy” (LEPERT), for patients with liver cancer. Moreover, we evaluate the dose difference of the conventional treatment planning with 10 MV X‐ray beam (MV‐plan) and LEPERT.

**Methods:**

The computed tomography (CT) was modeled with the Monte Carlo simulation. For LEPERT, 120 kV X‐ray beams collimated with CT were irradiated on a virtual tumor filled with Lipiodol at 10–50 mg/mL, which was inserted into a whole‐body phantom. A prescribed dose of 40 Gy/4fr was irradiated to achieve D_95%_ of the target. The doses to the target and organs at risk (OARs), such as the bone, normal liver, spinal cord, and kidneys, were evaluated by comparison with conventional radiotherapy with a 10 MV VMAT plan (MV‐plan).

**Results:**

Differences in the effective energy and off‐axis ratio between the measurements and simulations were within 2 keV and 3%, respectively. The D_2%_ of tumors exceeded 130% of the prescribed dose at 50 mg. The difference in the D_98%_ of the tumor between LEPERT and MV‐plan was within 0.7 Gy. The V_5Gy_ of the normal liver (>40 mg/mL) was lower for LEPERT than for MV‐plan. The V_20Gy_ of the normal liver (>10 mg/mL) for LEPERT was over 80% lower than that for MV‐plan. Dose constraints for the OARs were satisfied.

**Conclusion:**

The LEPERT can selectively enhanced only the tumor region with sparing the OAR dose. It could be a novel and effective treatment technique in the point that the treatment machine is a general CT device.

## 1 INTRODUCTION

Water‐based contrast media have been used to enhance lesion‐to‐tissue contrast during the computed tomography (CT) simulation used for radiotherapy treatment planning. However, the intravenous (IV) contrast is not used during treatment delivery, leading to density differences between the treatment planning CT and the treatment anatomy.^1^ While several studies have shown that contrast media have minimal influence on dose calculation for high‐energy photon beams, such as those ranging from 6 to 25 MV,^1^, ^2^, ^3^ dose enhancement effects have been observed when using low‐energy X‐rays with high‐Z materials, such as gold or iodine nanoparticles.^4^, ^5^, ^6^ The current study explores the possibility of selectively enhancing low‐energy radiation dose using a high atomic number (Z) contrast medium.

Preclinical studies used gold nanoparticles at a concentration within 6.0 mg/g.^4^, ^5^, ^6^ Cho et al. investigated the dosimetric effects of gold nanoparticles at 7 mg/g for kilovoltage X‐ray treatments.^7^ They showed that gold nanoparticles caused a dose enhancement of 200% upon irradiation with 140 kVp X‐rays. Gold nanoparticles (GNPs) exhibit a high absorption rate at low X‐ray energies, particularly between 10 and 50 keV, leading to a substantial increase in dose delivery to tumors. The dose enhancement effect is most pronounced in this energy range, with maximum dose enhancement factors (DEFs) observed, potentially increasing the delivered dose several‐fold.^4^ A water‐based contrast medium is absorbed and eliminated from the tumor or artery, which temporarily increases the contrast medium concentration. Additionally, an increase in dose enhancement is expected when using low‐energy photon beams in the contrast medium. Berger et al. showed that the energy at the boundary between the photoelectric effect and Compton scattering was higher for iodine (0.3 MeV) than for water (0.03 MeV).^8^ For the low‐energy photon beam, the photoelectric effect is dominant for iodine. Based on the dose enhancement for contrast medium, contrast‐enhanced radiation therapy (CERT), in which a low‐energy X‐ray beam (80–120 kVp) irradiates the contrast‐enhanced tumor region, has been developed.^9^ Iwamoto et al. investigated the feasibility of radiation dose enhancement therapy for contrast‐enhanced brain tumors in rabbits.^10^ They delivered 15 Gy of 120 kVp X‐rays in three fractions to a tumor enhanced with the contrast medium at a dose of 3.5 g of iodine per kg of body weight. The estimated dose enhancement was 30% based on CT scans. The median survival time after tumor detection by CT was 3 days for untreated rabbits, 25.5 days for those treated with radiation alone, and 38.5 days for those treated with radiation plus contrast media. Thus, contrast‐enhanced radiotherapy has shown promising results in vivo. On the other hand, studies on the increased bone dose due to low‐energy X‐rays have shown significant effects on bone marrow and adjacent normal tissues, potentially raising the absorbed radiation dose by up to 30% compared to higher‐energy photon sources.^11^, ^12^ For this risk, Iwamoto et al. suggested that normal tissue damage, particularly to skin, bone, and meninges can be reduced by the use of multiple fields for CERT.

Lipiodol (Guerbet, Villepinte, and France), an oil‐based contrast medium, has been used as an embolic agent in transarterial chemoembolization (TACE) to visualize tumors by CT. The washout rate of Lipiodol remaining in the tumor has a half‐life of 23.09 days.^13^ Radiotherapy after TACE has shown promising response.^14^, ^15^ A previous study evaluated the dose enhancement in Lipiodol with 10 MV flattening filter‐free (FFF) beams.^16^ The maximum dose enhancement with and without Lipiodol was 6.0%. In the build‐up region around Lipiodol, the enhancement was caused by a backscatter at the outer 3 mm of Lipiodol.^17^ Moreover, the dose enhancement was higher with the FFF beam than with a flattening filter (FF) beam.^18^ Thus, low‐energy photons and electrons contribute to dose enhancement, and hence, Lipiodol is a potential radiosensitizer.

**FIGURE 1:**
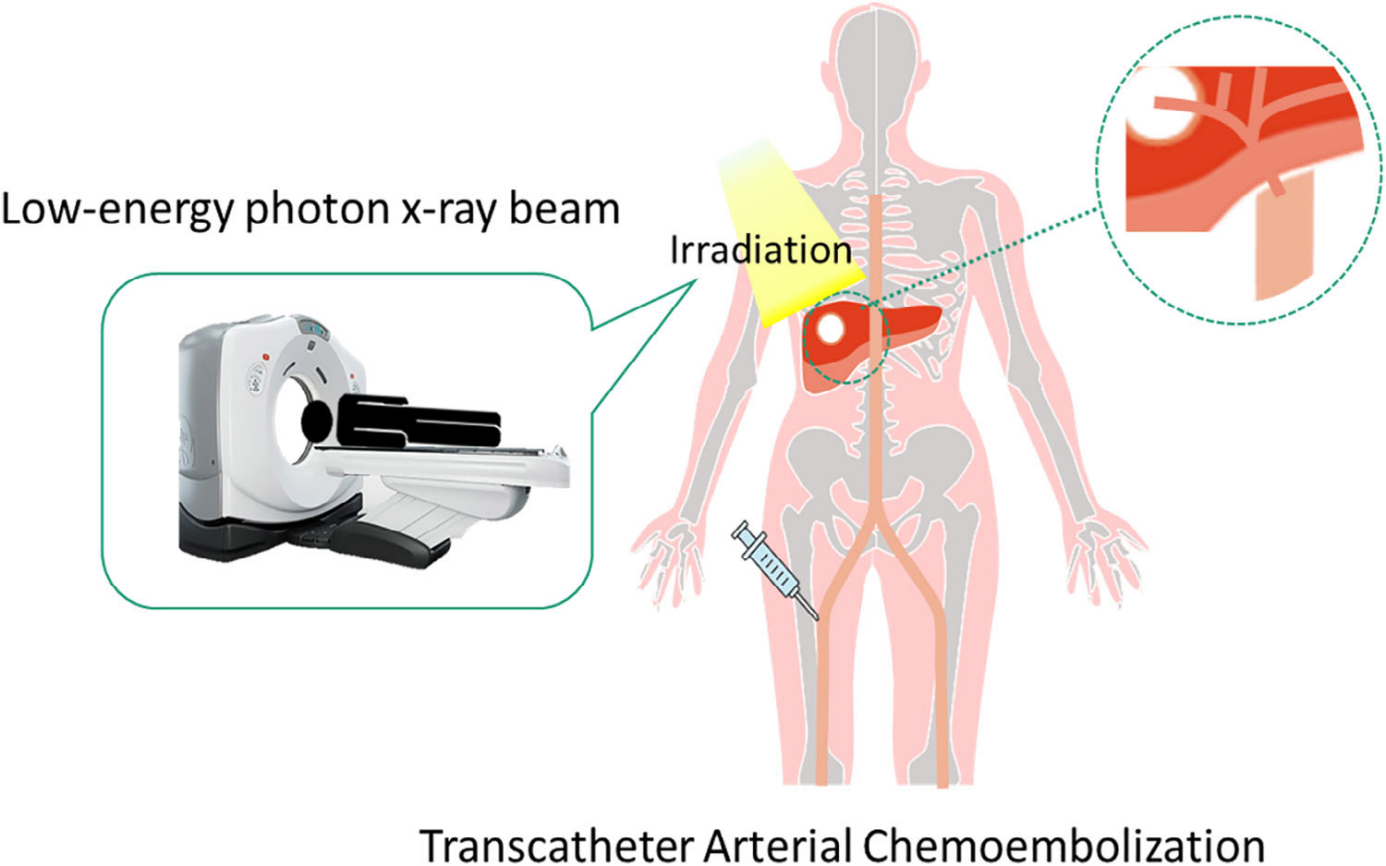
Schematic representation of the steps for the LEPERT. Lipiodol was filled in the tumor at TACE for tumor seeking on the CT. After TACE, the collimated 120 kV‐X ray beams with the CT were irradiated to the virtual tumor filled with Lipiodol.

Theoretically, the dose enhancement is larger with gold nanoparticles than with Lipiodol when the concentration in the tumor is the same. However, high concentrations of gold nanoparticles are toxic, although low concentrations do not affect cell viability.^19^ Compared to gold nanoparticles that are also used as dose‐enhancing media, Lipiodol is less toxic, and the concentrations do not affect cell viability. Lipiodol that is administered during TACE may be a viable a dose‐enhancing contrast agent during post‐TACE radiotherapy. Lipiodol has a lower toxicity profile than gold nanoparticles and has a long washout rate compared to water‐based contrast agents, and therefore does not require another injection after TACE is performed. The current study proposes a novel low‐energy photon lipiodol‐enhanced radiotherapy (LEPERT) for patients with hepatocellular carcinoma, as shown in Figure 1. Moreover, we evaluated the dose difference between conventional treatment planning using a 10 MV X‐ray beam (MV‐plan) and LEPERT.

## 2 METHODS AND MATERIALS

### 2.1 Monte Carlo simulation modeling with low‐energy photon beam

**FIGURE 2:**
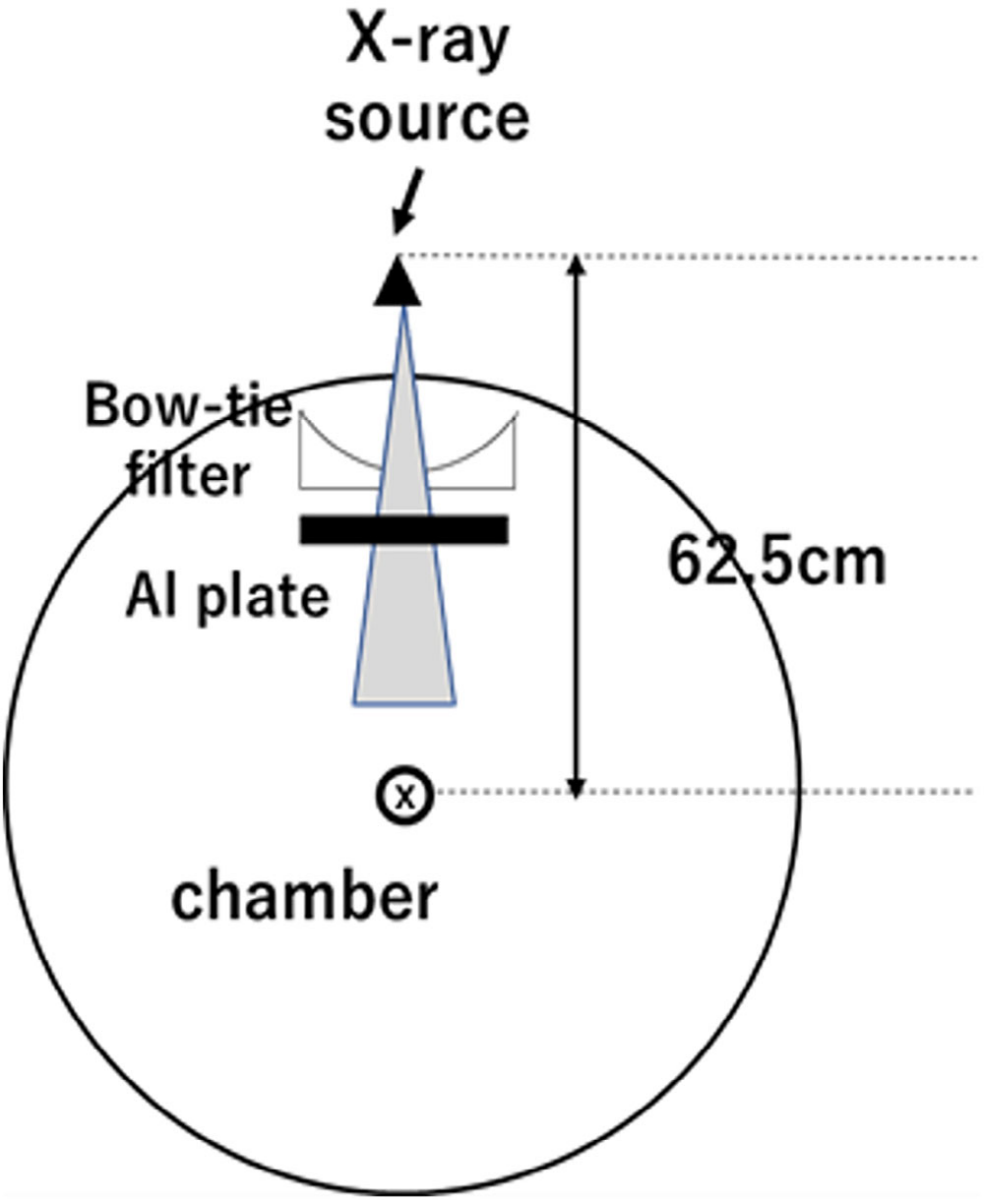
The experimental setup of the HVL measurement.

A Revolution CT scanner (GE Healthcare, Princeton, New Jersey, USA) was modeled using the Monte Carlo code BEAMnrc. The inherent filter material was titanium, and the bowtie filter was Teflon. The minimum and maximum thicknesses of the bowtie filter were 0.15 and 2.8 cm, respectively. The CT scanner had a focal spot‐to‐axis distance of 62.56 cm. The tube voltage and current were set to 120 kVp and 150 mA, respectively. The dose calculations were performed using DOSXYZnrc. The numbers of photon histories in BEAMnrc and DOSXYZnrc were 2.0 × 10^8^ and 2.0 × 10^9^, respectively. The statistical error in the calculation was within 1.0%. The photon and electron cut‐off energies were set to 0.001 and 0.516 MeV, respectively, based on values reported in previous literature. These cut‐off values were chosen to ensure a balance between computational efficiency and accuracy, as higher cut‐off energies may lead to information loss in low‐energy interactions, while lower values can significantly increase computation time.^20^ The Monte Carlo calculation was validated using the effective energy and off‐center ratio (OCR). Figure 2 shows the experimental set‐up for these measurements. Aluminum half‐value layer (AL HVL) and OCR were measured at the isocenter of the scanner using a 0.6 cc farmer‐type air ionization chamber (30013, PTW). The amount of charge was measured by varying the thickness of the aluminum plate while keeping the tube stationary. The HVL was measured for a tube voltage of 120 kV in CT. The effective energies were calculated using basic data from Selzer and Hubbell.^21^

The dose enhancement was validated by comparing the measurements and calculations with the results from the Monte Carlo simulation. Measurement data were obtained using a GAFchromic film.^21^, ^22^ A 120 kVp X‐ray beam was directed toward a contrast medium containing 100 mg/mL of Lipiodol (Optiray, Mallinckrodt) positioned behind a 4.8 cm PMMA slab phantom. The source‐to‐surface distance (SSD) was 15 cm.

### 2.2 Concentration of Lipiodol for clinical patients

Ten patients treated with radiotherapy after TACE between 2015 and 2018 were selected, and the patients who did not washout were eliminated by comparison with those immediately after TACE. The CT value in the gross tumor volume (GTV) on treatment planning CT was measured using the Eclipse treatment planning system, version 16.1 (Varian Medical Systems, USA). A conversion curve of the CT value to the contrast medium concentration was plotted. The Lipiodol concentration was calculated using the conversion curve.

### 2.3 Lipiodol‐enhanced radiotherapy (LEPERT)

**FIGURE 3:**
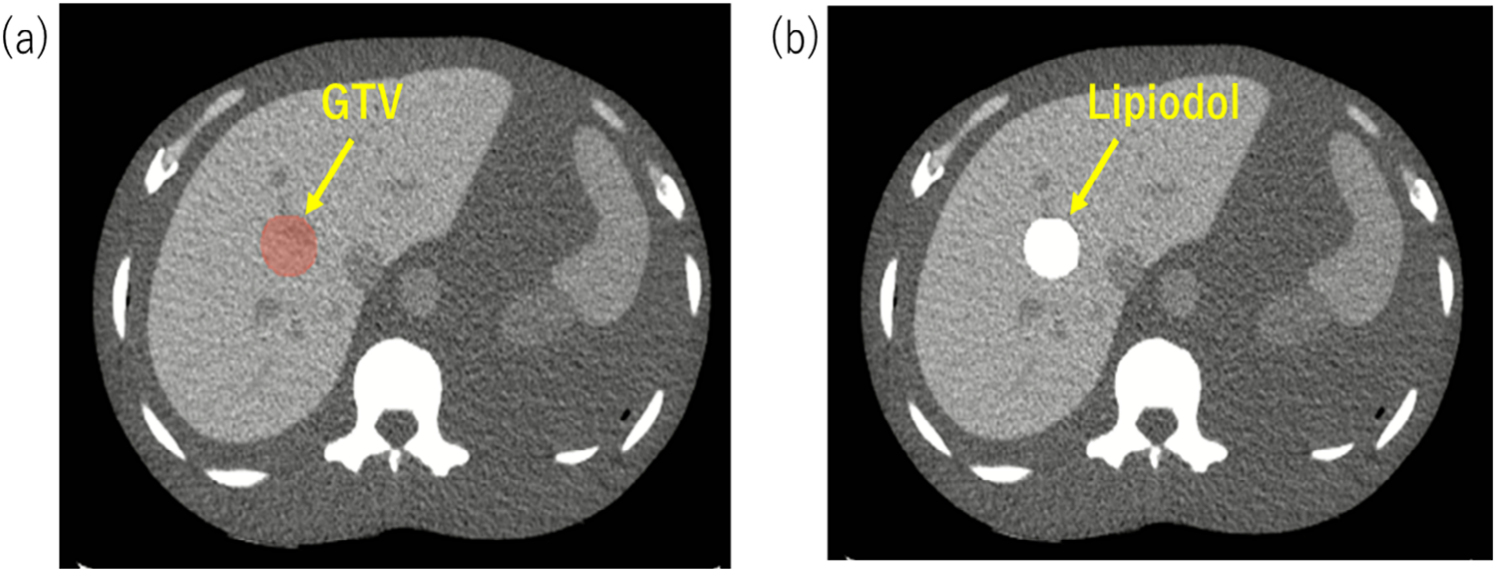
(a) Virtual tumor region of a 3 cm sphere defined as the GTV on the CT image. (b) Lipiodol region assigned to 10 000 HU in the GTV.

An anthropomorphic whole‐body phantom (PBU‐60; Kyoto Kagaku Co. LTD, Kyoto, Japan) was scanned using CT (LightSpeed RT16; GE Healthcare, Little Chalfont, UK). The slice thickness and interval were 1.25 mm, and the tube voltage was 120 kVp. The scanned CT images were transferred to a medical image computing tool called 3D Slicer (www.slicer.org, accessed on January 1, 2020).^23^ The virtual tumor region of a 3 cm sphere was defined as the GTV (Figure 3a). A planning target volume (PTV) margin of 5 mm is typically added. The generated CT images and structural data were transferred to our in‐house system in MATLAB to create binary data files containing the voxelized information in a format that is easily input into the Monte Carlo calculation. The translated data were read into an array containing an element for each voxel in the structure dataset and original CT. These data preserve the structural data, spatial location of the original CT voxel, and HU. For material assignment, the GTV region was assigned to the Lipiodol with 10–50 mg/mL (Figure 3b). The CT image in the other region was assigned using our own CT calibration curve, determined using a Gammex 467 tissue characterization phantom (Gammex, Inc., Middleton, Wisconsin, USA). The materials of the voxel‐based CT phantom were selected to convert the CT images into a media map (air, lung, soft tissue, and bone) and mass density. The beam collimation was performed with a rectangular field covering the PTV. A dose of 40 Gy in four fractions was prescribed to the D_95%_ of the GTV.

### 2.4 Conventional high‐energy X‐ray radiotherapy treatment planning

The conventional high‐energy X‐ray radiotherapy treatment planning was created using linear accelerator and treatment planning system (RTPS). A TrueBeam linear accelerator (Varian Medical Systems, Palo Alto, California, USA) was used to provide 10 MV FFF beams. The RTPS used Eclipse version 16.1 (Varian Medical Systems, Palo Alto, USA) with the Acuros XB (AXB) algorithm. The material composition of a given voxel in a 3D CT image was automatically assigned from the CT value based on our own CT calibration curve determined using the Gammex 467 tissue characterization phantom (Gammex, Inc., Middleton, Wisconsin, USA) and material data in the AXB algorithm. The materials in the AXB algorithm were assigned automatically as one of the following: “air,” “lung,” “adipose tissue,” “skeletal muscle,” “cartilage,” and “bone.” The materials were assigned according to the range of CT values. In the conventional high‐energy X‐ray radiotherapy treatment planning (MV‐plan), the tumor region was not assigned to Lipiodol. A previous study reported that when Lipiodol was assigned to the “bone” in the material assignment of the AXB algorithm, the Lipiodol region caused a dose decrease.^10^ Thus, the MV‐plan assumed that Lipiodol was not assigned to a voxel phantom. The Lipiodol region was assigned to the “Water.” A volumetric‐modulated radiotherapy (VMAT) plan was created using coplanar beams. The gantry angle was set to 180°–0° in the clockwise direction. The couch and collimator angles were fixed at 0° and 10°, respectively. The field size was 4 × 4 cm^2^, which a 1 cm margin was added from the target. A dose of 40 Gy in four fractions was prescribed to the D_95%_ of the PTV. The dose constraints for other organs at risk (OARs) were obtained from a previous study and are presented in Table S1. ^24^ The doses to the target and OARs, such as the bone, normal liver, spinal cord, and kidneys, were evaluated.

## 3 RESULTS

### 3.1 Monte Carlo simulation modeling with low‐energy photon beam

**FIGURE 4:**
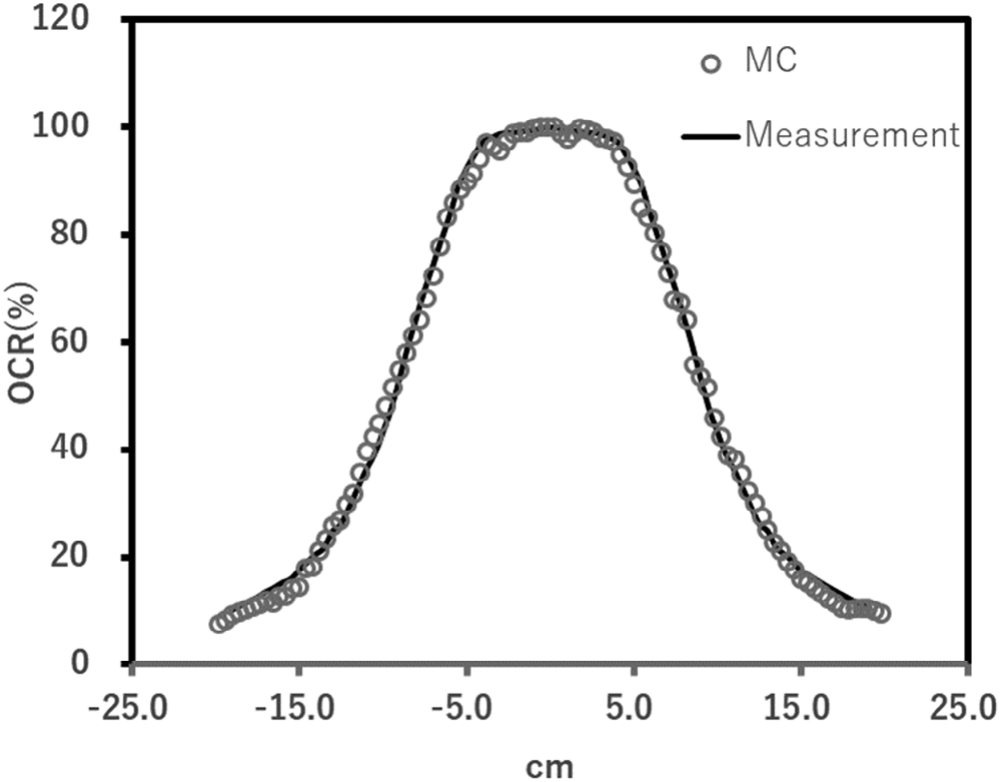
Comparison of OCR of the MC calculation and measurement.

Table 1 lists the Al HVLs and effective energies for the measurement and calculation. The differences in the Al HVL and effective energy were within 0.2 mm and 2 keV, respectively. Figure 4 shows a comparison of the OCR between the measured and calculated results. The measurement and simulation results are within 3%.

**TABLE 1:** AL HVLs and effective energies for the measurement and Monte Carlo calculation.

	Measurement	MC
HVL (mm)	9.2	9.4
Effective energy (keV)	59	61

**FIGURE 5:**
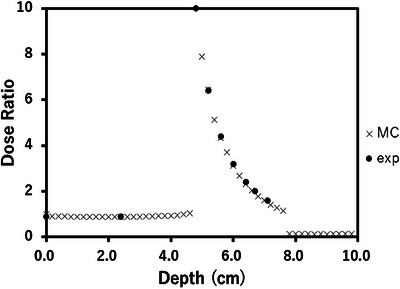
Comparison of the measurement (exp) with the radiochromic film^25^ and MC‐calculated dose enhancement in a contrast medium containing 100 mg/mL of iodine (Optiray, Mallinckrodt) positioned behind a 4.8 cm PMMA slab phantom with 120 kVp X‐rays generated by the CT scanner. The source‐to‐surface distance (SSD) was 15 cm and the contrast medium positioned behind a 4.8 cm PMMA slab phantom. A high Pearson correlation coefficient (γ = 0.999) between the measured and calculated dose enhancement values further supports the close agreement between experimental measurements and MC simulations within the contrast medium.

Figure 5 shows the results for calculation and measurement^25^ of the dose enhancements with a 100 mg/mL contrast medium, irradiated by 120 kVp photons. As shown in Figure 5, a very high correlation was observed between the measured dose enhancement data and the MC simulation results with 120 kVp X‐rays. The Pearson correlation coefficient (γ) between the measured and calculated values was 0.999 within the contrast medium, confirming the high accuracy of the MC simulation. The difference between the measured and calculated values was within 2.3%. Dose enhancement was confirmed through both measurements and calculations. Measurements with the contrast medium and MC calculations with Lipiodol were within 3%.

### 3.2 Concentration of the Lipiodol for clinical patients

**FIGURE 6:**
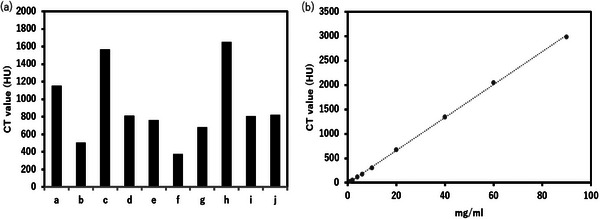
The mean concentration of the Lipiodol on the CT image for clinical patients (a) and relation of the Lipiodol concentration and CT value (b). The Pearson correlation coefficient (γ = 0.999) between Lipiodol concentration and CT values demonstrates the strong linear relationship.

Figure 6 shows the mean concentration of Lipiodol on the CT images of clinical patients and relationship between the Lipiodol concentration and CT value. The relationship between Lipiodol concentration and CT values showed a Pearson correlation coefficient (γ) of 0.999, indicating that CT values provide a reliable estimate of concentration. The relationship between the concentration of the contrast medium and CT value was obtained using an acrylic phantom containing inserts with different concentrations of iodinated contrast medium, as used in a previous study. The CT scans were performed at a tube voltage of 120 kVp. The minimum and maximum CT value were 376.2 and 1653 HU, respectively, corresponding to concentrations of 11.4 and 49.4 mg/mL, respectively. The mean CT value was 864.7 HU, corresponding to a concentration of 26.0 mg/mL.

### 3.3 Lipiodol‐enhanced radiotherapy (LEPERT)

**FIGURE 7:**
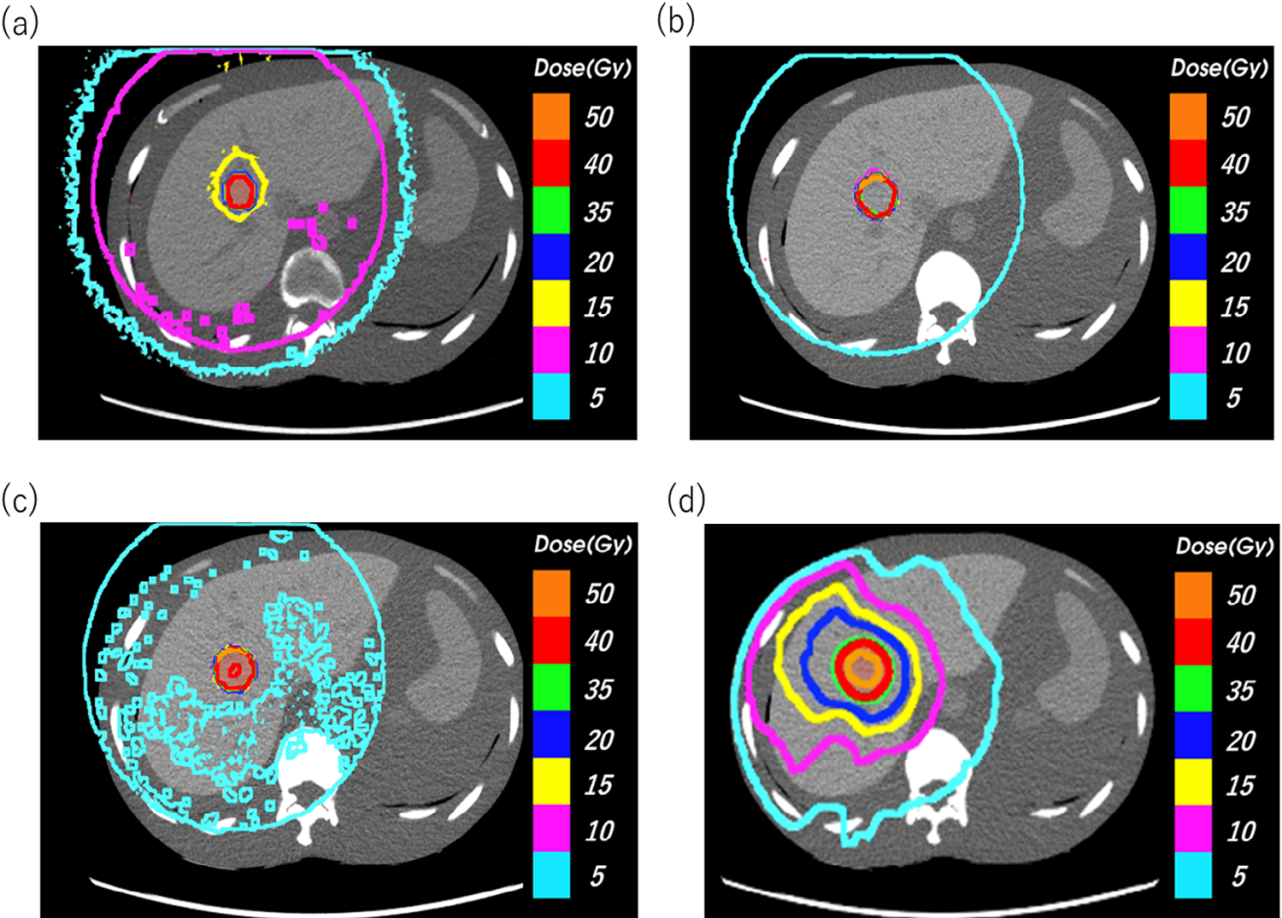
Dose distribution at the axial plane in LEPERT at concentrations of (a) 10 mg/mL, (b) 30 mg/mL, and (c) 50 mg/mL. (d) Conventional high‐energy X‐ray radiotherapy treatment planning (MV‐plan).

**FIGURE 8:**
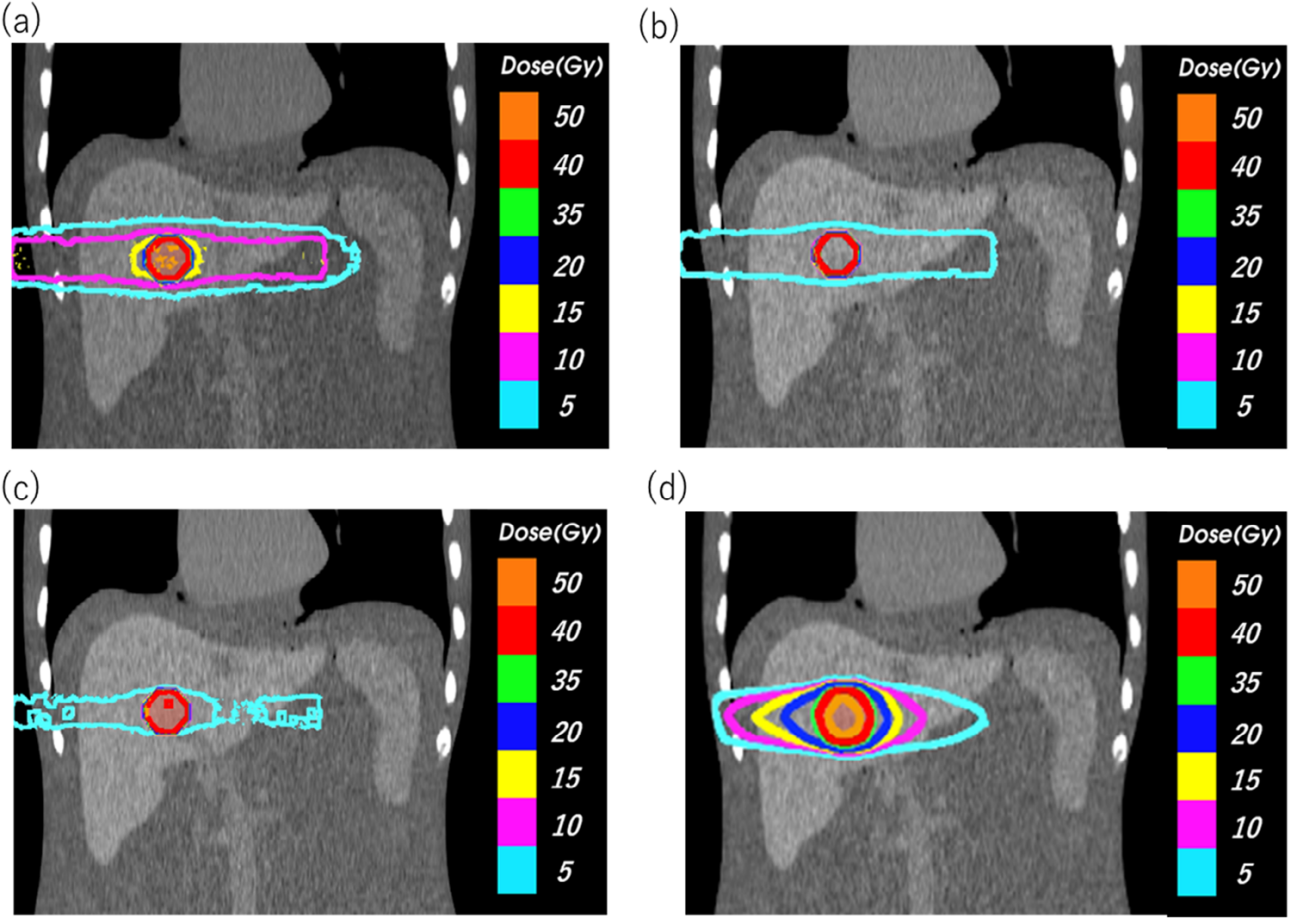
Dose distribution at the coronal plane in LEPERT at concentrations of (a) 10 mg/mL, (b) 30 mg/mL, and (c) 50 mg/mL. (d) Conventional high‐energy X‐ray radiotherapy treatment planning (MV‐plan).

Figure 7a–c show the dose distribution at the axial plane in LEPERT, and Figure 7d shows the dose distribution at the axial plane in the MV‐plan. Figure 8a–c show the dose distribution at the coronal plane in LEPERT, and Figure 8d shows the dose distribution at the coronal plane in the MV‐plan. For LEPERT at 30 and 50 mg/mL, an isodose line of exceeding 15 Gy was focused on the tumor compared to that at 10 mg/mL and the MV‐plan. The isodose line at 5 Gy shrank with higher concentrations of Lipiodol. The isodose line at 5 Gy was reduced for LEPERT at 30 and 50 mg/mL when compared with the MV‐plan.

**FIGURE 9:**
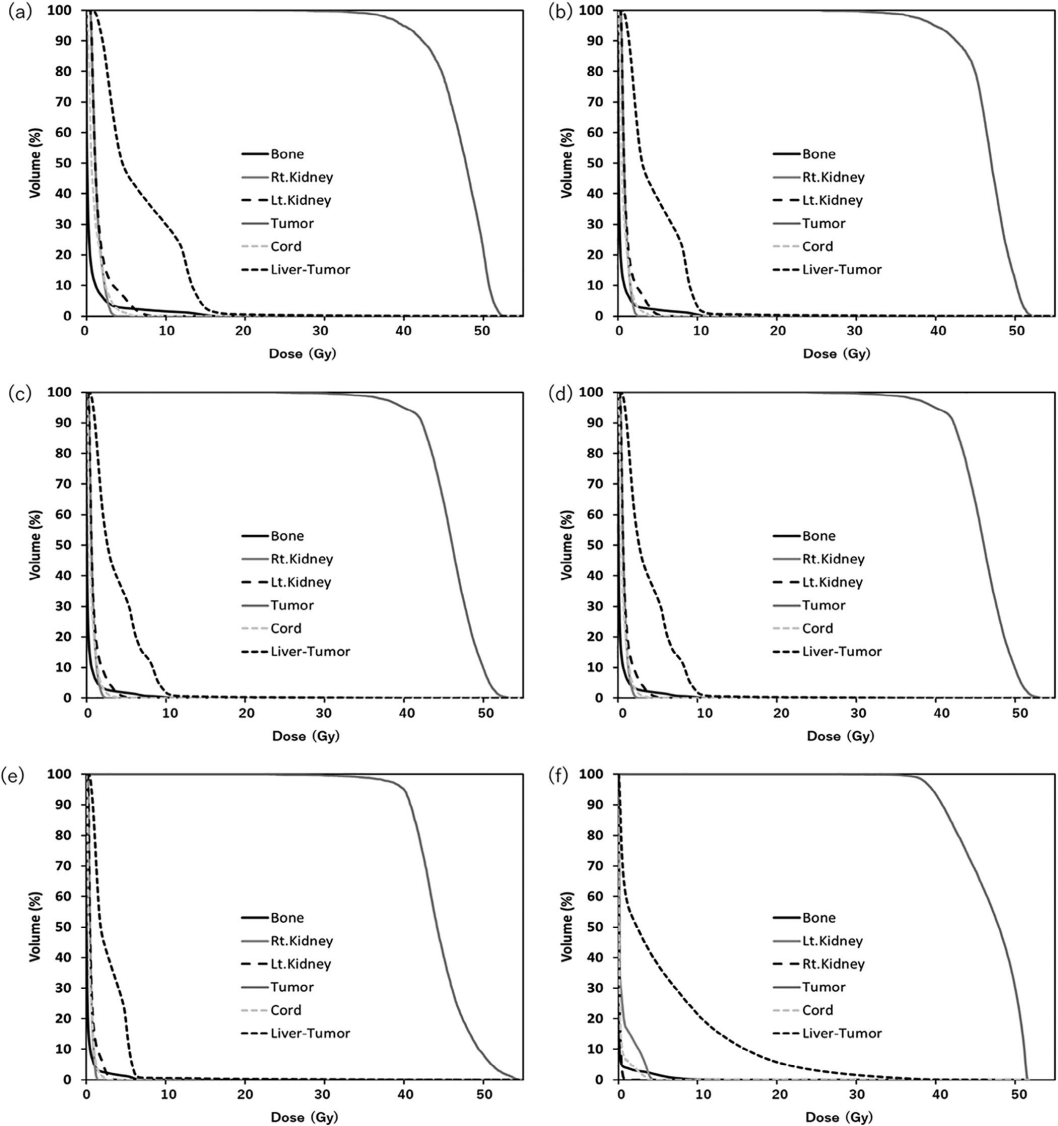
Dose volume histogram (DVH) for the target and organ sat risk (OARs) such as bone, normal liver, and spinal cord in LEPERT at concentrations of (a) 10 mg/mL, (b) 20 mg/mL, (c) 30 mg/mL, (d) 40 mg/mL, and (e) 50 mg/mL. (f) Conventional high‐energy X‐ray radiotherapy treatment planning (MV‐plan).

Figure 9a–e show the dose volume histogram (DVH) of the target and OARs, such as the bone, normal liver, and spinal cord, in LEPERT at concentrations of 20 and 40 mg/mL. Figure 9f shows the DVH of the target and OARs in the MV‐plan. Table 2a summarizes the results of the DVH numerical analysis, including the *D*_98%_ (dose encompassed by 98% volume), *D*_50%_, *D*_2%_, and *D*_mean_ of the target for LEPERT at concentrations of 10–50 mg/mL, as well as the MV‐plan. The *D*_98%_ of the tumors with LEPERT were smaller than those with the MV‐plan. The *D*_50%_ of the tumors increased with increasing concentrations of Lipiodol. The *D*_50%_ and *D*_mean_ of the tumor treated with LEPERT was higher than that of the tumor treated with the MV‐plan for concentrations below 30 and 40 mg/mL, respectively. The *D*_2%_ and *D*_mean_ of the tumor decreased with higher concentrations of Lipiodol. The *D*_2%_ of the tumors treated with LEPERT at a concentration of 50 mg/mL exceeded 130% of the prescribed dose. Table 2b presents the results of DVH numerical analysis for a normal liver. The *V*_5Gy_, *V*_20Gy_, and *D*_mean_ values of the normal liver decreased with higher Lipiodol concentrations in LEPERT. The volume of the normal liver by the 5 Gy isodose line (*V*_5Gy_) in LEPERT was smaller than that in the MV‐plan for concentrations above 30 mg/mL. The *V*_20Gy_ of the normal liver with LEPERT was significantly lower than that with the MV‐plan at all concentrations. The *D*_mean_ of the normal liver with LEPERT was smaller than that with the MV‐plan by more than 20 mg/mL. Table 2c presents the results of the DVH numerical analysis of the other OARs, namely the bone, spinal cord, and right and left kidneys. The *D*_0.1cc_ of the bone, spinal cord, and left and right kidneys with LEPERT was smaller with higher concentrations of Lipiodol. The *D*_0.1cc_ of bone with LEPERT was smaller than that with the MV‐plan at concentrations exceeding 20 mg/mL. The *D*_0.1cc_ of the spinal cord with LEPERT was smaller than that with the MV‐plan at concentrations exceeding 40 mg/mL. The *D*_mean_ of the left and right kidneys with LEPERT was smaller than that with the MV‐plan at concentrations exceeding 30 mg/mL.

**TABLE 2:** Results of DVH numerical analysis of the (a) tumor, (b) normal liver, and (c) other OARs.

(a) Tumor	10 mg/mL	20 mg/mL	30 mg/mL	40 mg/mL	50 mg/mL	MV‐plan
*D*_98%_ (cGy)	37.3	36.8	36.8	36.9	37.4	37.5
*D*_50%_ (cGy)	47.8	47.0	46.0	44.9	44.2	46.3
*D*_2%_ (cGy)	51.8	51.3	51.6	51.9	52.6	51.1
*D*_mean_ (cGy)	37.7	37.2	36.6	36.0	35.5	36.0

(b) Normal liver	10 mg/mL	20 mg/mL	30 mg/mL	40 mg/mL	50 mg/mL	MV‐plan
*V*_5Gy_ (%)	47.1	38.2	32.4	26.6	18.6	37.6
*V*_20Gy_ (%)	0.5	0.3	0.3	0.3	0.3	5.74
*D*_mean_ (%)	540.2	367.6	308.5	249.2	222.7	367.7

(c) Other OARs	10 mg/mL	20 mg/mL	30 mg/mL	40 mg/mL	50 mg/mL	MV‐plan
Bone: *D*_0.1cc_ (cGy)	18.4	12.4	11.1	9.8	7.4	14.1
Spinal cord: *D*_0.1cc_ (cGy)	15.5	10.7	8.9	7.2	6.4	7.8
Right kidney: *D*_mean_ (%)	2.6	1.8	1.5	1.2	1.0	1.3
Left kidney: *D*_mean_ (%)	3.2	2.2	1.8	1.5	1.3	2.1

## 4 DISCUSSION

The results from MC calculations of the absorbed dose from low‐energy x‐rays delivered to a phantom with a Lipiodol insert show that Lipiodol can be used as a radiosensitizer with low‐energy x‐rays. To ensure the validity of the in‐phantom calculations, the MC algorithm was validated for Lipiodol with measurements. Agreements of 3% were observed under validation conditions. Currently, high‐energy photon radiotherapy is performed after liver TACE; however, the results show that it would be possible to use the TACE‐contrast agent, Lipiodol, as a tumor radiosensitizer with low‐energy x‐rays, since the dose enhancement is up to 330% for low‐energy x‐rays compared to 6% for the high‐energy treatments.^16^ The study evaluated the average concentration of Lipiodol after TACE, and the calculations were performed on the observed clinical concentrations. The washout rates of Lipiodol are such that the medium will be present in the radiotherapy setting. As such, these results are expected to be clinically applicable.

Verhaegen et al. simulated the CERT for brain tumors using a CT scanner.^25^ A contrast‐enhanced target with a concentration of 50 mg/mL, representing a spherical tumor with a diameter of 3 cm, was inserted into the cylinder. Dose enhancement exceeded 400% in the tumor region. However, the concentration of the contrast medium in the clinical setting was not reflected. The concentrations of the contrast medium and Lipiodol are major factors influencing dose distribution and treatment outcome. Melo et al. investigated the concentration of contrast medium in brain tumors.^10^ They injected 1–2 mL of contrast material (CM) per kg, which reached 5 mg/mL iodine in the tumor region. Sahbaee et al. estimated the radiation dose enhancement with a contrast medium in a CT examination.^2^ The results of dose enhancement from the patient models showed that 35% of the radiation dose was delivered to the liver. The concentration of the contrast medium in the liver was below 4 mg/mL. Thus, conventional low‐energy radiotherapy could not provide a higher dose to the tumor in the clinical situation. On the other hand, the Lipiodol is directly delivered to the tumor through arteries. Additionally, the concentration remained high owing to the prevention of Lipiodol washout by transient embolization. The average concentration of the Lipiodol after TACE remained 26.0 mg/mL in the tumor region. The use of low‐energy X‐rays in LEPERT represents a groundbreaking treatment method, allowing for highly localized dose delivery to the tumor.

The *D*_mean_ of the bilateral kidneys was sufficiently low with both the MV‐plan and LEPERT at concentrations of 10–50 mg/mL. For LEPERT at a concentration of 10 mg/mL, the *V*_5Gy_ for the normal liver and D0.1cc for the bone and spinal cord were higher than those for the MV‐plan and satisfied the dose constraints. LEPERT at a concentration of 20 mg/mL had a smaller *V*_20Gy_ of the normal liver and D0.1cc of the bone than the MV‐plan. The difference in the V5Gy of the normal liver for LEPERT at 20 mg/mL was 0.6% higher than that of the MV‐plan. In contrast, LEPERT at a concentration of 30 mg/mL had a smaller *V*_5Gy_ and *V*_20Gy_ of the normal liver and D_0.1cc_ of the bone. For the LEPERT at a concentration of 50 mg/mL, the OAR dose was significantly smaller. Thus, LEPERT at concentrations exceeding 20 mg/mL outperformed the MV‐plan in many DVH metrics, and can be a good or better treatment than conventional photon therapy. LEPERT could significantly improve treatment efficacy and minimize damage to surrounding healthy tissues by exploiting the high absorption rates of low‐energy X‐rays in the presence of Lipiodol.

This study had several limitations. First, the current study did not evaluate the thermal loading restriction associated with using conventional CT scanners for radiotherapy. Delivering the necessary treatment dose to human patients may be challenging due to these thermal constraints. Previous studies, including those by Rose et al., have addressed this issue by improving the heat capacity of CT scanners, allowing for repeated high‐dose fractions without overheating.^26^ The modified CT scanner used in this research demonstrated enhanced thermal management, which is promising for safely delivering therapeutic radiation doses while maintaining operational stability. Moreover, the uptake of Lipiodol may not be uniform in the tumor and could differ from case to case. CT images were obtained immediately after TACE and during follow‐up sessions, suggesting that the washout rates of Lipiodol could be minimized by administering LEPERT immediately after TACE. Finally, while this study is grounded in simulations and calculations, further research is essential to evaluate the radiobiological effects by comparing in vivo and in vitro results with the predictions made in this study. It is important to emphasize that this work represents a conceptual exploration rather than a clinical protocol, and thus, radiobiological or clinical responses have not been assessed at this stage.

## 5 CONCLUSION

LEPERT can selectively enhance the tumor region, while sparing the OAR dose. This could be a novel and effective treatment technique because the treatment machine is a general CT device that can be combined with TACE.

A proposed “Low‐energy photon Lipiodol‐Enhanced Radiotherapy” (LEPERT) can selectively enhanced only the tumor region with sparing the OAR dose. It could be a novel and effective treatment technique in the point that the treatment machine is a general CT device.

## AUTHOR CONTRIBUTIONS

This study was performed by only a single researcher (Daisuke Kawahara). Daisuke Kawahara proceeded with the overall research and wrote an article.

## CONFLICT OF INTEREST STATEMENT

The author declares no conflicts of interest.

## Supporting information



Supporting information
