# Supplementary material for: Enhancing targeted doses: Low‐energy photon lipiodol‐enhanced radiotherapy (LEPERT) for liver cancer patients
Source: J Appl Clin Med Phys. 2024 Nov 29;26(3):e14578. doi: 10.1002/acm2.14578 (PMC11905249; doi:10.1002/acm2.14578)
Supplement: Supplementary file 1 — Supporting information [file ACM2-26-e14578-s001.docx]

Table S1. Dose constraints of planning organ at risk volume.

| **PRV** | **Volume** | **Dose constraints** | **Max dose** |
| --- | --- | --- | --- |
| Spinal cord | <0.1 cc | 22 Gy | 28 Gy |
| Liver | <60 % | 5 Gy |  |
|  | <20 % | 20 Gy |  |
